# Primary hepatic gastrinoma being diagnosed preoperatively: a case report and literature review

**DOI:** 10.1186/s40792-020-01072-9

**Published:** 2020-11-18

**Authors:** Shunya Hanzawa, Hiroshi Sadamori, Masaaki Kagoura, Kazuteru Monden, Masayoshi Hioki, Tsuyoshi Hyodo, Kunihiro Omonishi, Norihisa Takakura

**Affiliations:** 1 Department of Surgery, Fukuyama City Hospital, 5-23-1 Zao-cho, Fukuyama, Hiroshima 721-8511 Japan; 2 Department of Radiology, Fukuyama City Hospital, 5-23-1 Zao-cho, Fukuyama, Hiroshima 721-8511 Japan; 3 Department of Pathology, Fukuyama City Hospital, 5-23-1 Zao-cho, Fukuyama, Hiroshima 721-8511 Japan

**Keywords:** Primary hepatic gastrinoma, Zollinger–Ellison syndrome, Selective arterial calcium injection test, Somatostatin receptor scintigraphy

## Abstract

**Background:**

A majority of gastrinomas causing Zollinger–Ellison syndrome are located in the duodenum or pancreas. Primary hepatic gastrinomas are rare and difficult to diagnose. We report a rare case of primary hepatic gastrinoma, which could be diagnosed preoperatively.

**Case presentation:**

A 29-year-old man with a 55-mm tumor in segments 5 and 6 (S 5/6) of the liver was admitted to our hospital. After thorough investigations, he was treated for a suspected inflammatory pseudotumor and advised to undergo routine follow-up. Two years later, he revisited our hospital with a complaint of abdominal pain, vomiting, and diarrhea. Upper gastrointestinal endoscopy revealed multiple duodenal ulcers. His serum gastrin level was 2350 pg/mL (normal: 37–172 pg/mL), suggesting Zollinger–Ellison syndrome. Abdominal computed tomography showed a 78-mm hypervascular tumor with cystic degeneration in the S 5/6 region of the liver, with a potential to increase over time. The tumor showed hypointensity on T2-weighted and hyperintensity on diffusion-weighted abdominal contrast-enhanced magnetic resonance imaging. Somatostatin receptor scintigraphy (SRS) only detected a hepatic tumor. No tumors in the gastrinoma triangle were detected by endoscopic ultrasonography. Hence, selective arterial calcium injection (SACI) test was performed to determine the location of the gastrinoma. The serum gastrin concentration increased from 4620 pg/mL to 23,600 pg/mL at 20 s after calcium gluconate injection into the proper hepatic artery. Conversely, no effect on serum gastrin level was observed after the injection into any other arteries. Extended right hepatic lobectomy and cholecystectomy were performed after percutaneous transhepatic portal vein embolization. A histopathological examination of the liver tumor revealed a gastrinoma. The patient’s serum gastrin concentration on postoperative day 1 decreased to 65 pg/mL.

**Conclusion:**

We report a surgical case of primary hepatic gastrinoma correctly diagnosed preoperatively. The patient underwent extended right hepatic lobectomy, resulting in a histological definitive diagnosis of primary hepatic gastrinoma.

## Background

Gastrinomas are the most common pancreatic neuroendocrine tumors, and most of them cause Zollinger–Ellison syndrome (ZES) [1]. A majority of gastrinomas are located within the gastrinoma triangle that is bounded by the confluence of the cystic and common bile ducts, the second and third portions of the duodenum, and the head and tail of the pancreas [2].

Primary hepatic gastrinoma is rare and the diagnosis is often difficult to perform preoperatively. In most cases, the diagnosis could be made postoperatively by histological examinations in resected specimen. The exclusion of both the presence of tumor in the gastrinoma triangle and the possibility of being a metastasis from other intra-abdominal organs are necessary, using multiple modalities, such as upper gastrointestinal endoscopy, computed tomography (CT), somatostatin receptor scintigraphy (SRS), and selective arterial calcium injection (SACI) test.

In this paper, we report a rare case of primary hepatic gastrinoma that could be diagnosed preoperatively in a patient who underwent liver resection, resulting in a histological definitive diagnosis.

## Case presentation

A 29-year-old man with a 55-mm tumor in segments 5 and 6 (S 5/6) of the liver was admitted to our hospital. After thorough investigations, a malignant tumor could not be ruled out, and the plan was to perform an operation on the patient. However, a month later, the CT showed a tendency for the tumor to shrink, which is suggestive of a pseudo-inflammatory tumor. Thus, we suspected that his tumor was an inflammatory pseudotumor and advised him to undergo routine follow-up CT. Two years later, he revisited our hospital with a complaint of abdominal pain, vomiting, and diarrhea. We performed an upper gastrointestinal endoscopy and noted multiple duodenal ulcers. His serum gastrin level was 2350 pg/mL (normal range: 37–172 pg/mL), which was suggestive of ZES.

**Fig. 1:**
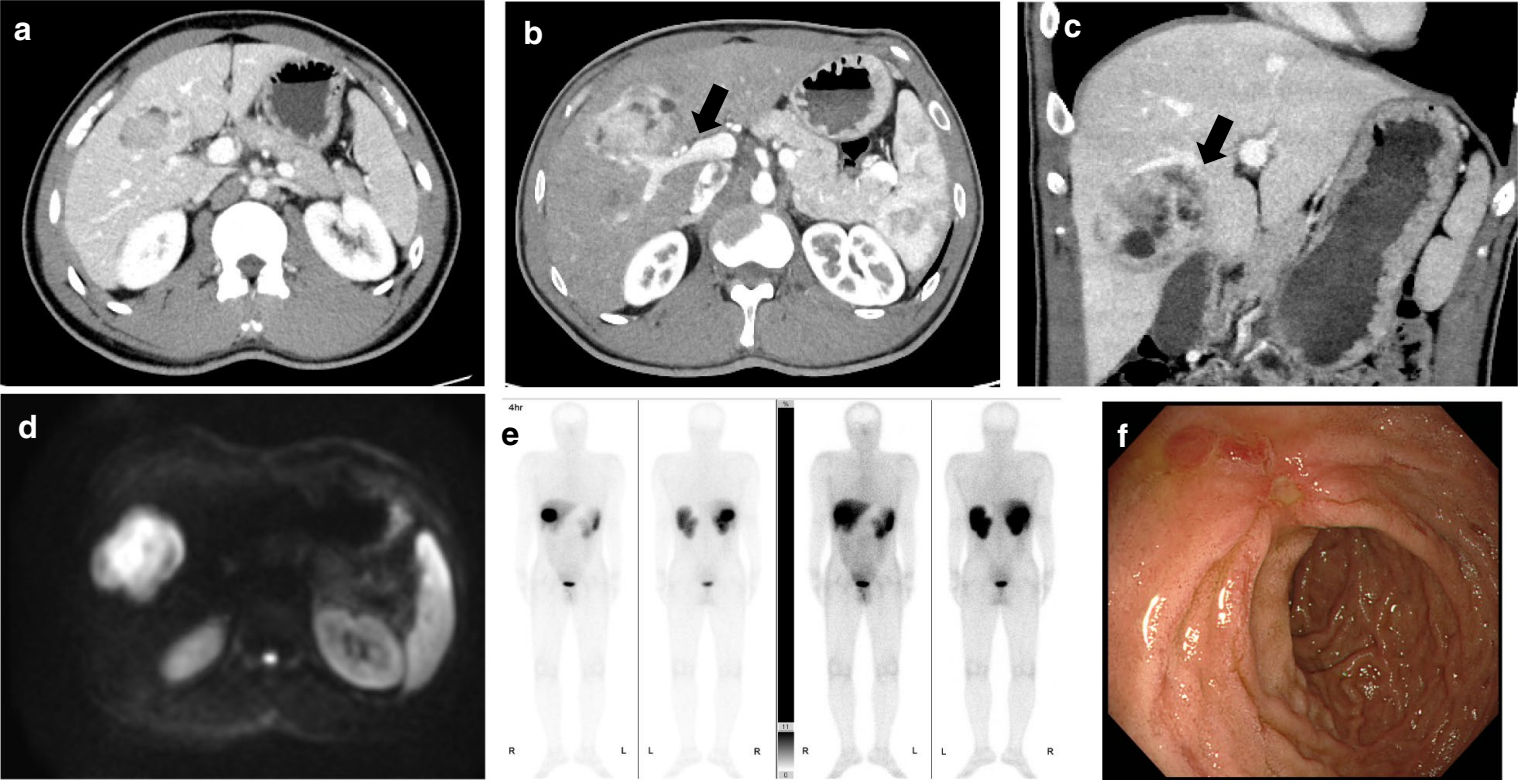
Preoperative imaging. **a** Abdominal contrast-enhanced computed tomography (CT) showed a tumor at the first visit (55 mm). **b** Abdominal contrast-enhanced CT showed a hypervascular tumor with cystic degeneration in segment 5/6 of the right hepatic lobe (78 mm). The tumor is in close proximity to the right branch of the portal vein (arrow). **c** The tumor partly involved the S4a region, and invasion to the middle hepatic vein (MHV) was suspected (arrow). **d** Magnetic resonance imaging (MRI) showed a hyperintensity on diffusion-weighted imaging. **e** Somatostatin receptor scintigraphy (SRS) showed abnormal uptakes in the liver. **f** Upper gastrointestinal endoscopy showed multiple duodenal ulcers (H1 stage)

Given that the patient was suspected of having a metastatic gastrinoma from other intra-abdominal organs, we performed several imaging studies, such as abdominal contrast-enhanced CT, magnetic resonance imaging (MRI), SRS, and SACI test. Abdominal CT showed a 78-mm hypervascular tumor with cystic degeneration in the S 5/6 region of the liver, with a potential to increase over time (Fig. 1a–c). The tumor showed hyperintensity on diffusion-weighted imaging using abdominal contrast-enhanced MRI (Fig. 1d). On SRS, a strong accumulation was found on the hepatic tumor (Fig. 1e). We performed endoscopic ultrasonography, but were unable to identify any tumors in the gastrinoma triangle. We detected multiple duodenal ulcers (H1 stage) by upper gastrointestinal endoscopy (Fig. 1f).

**Fig. 2:**
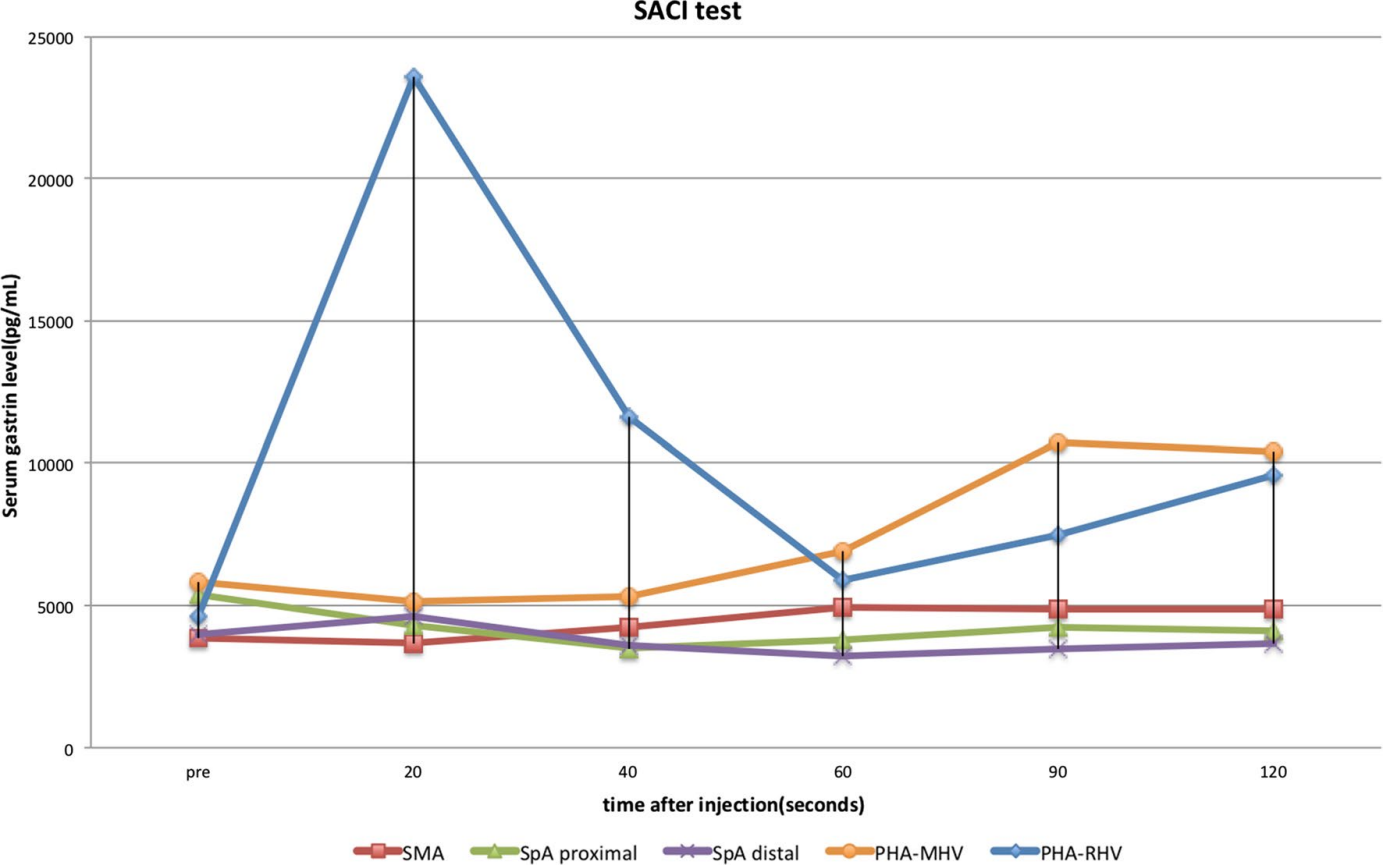
The selective arterial calcium injection (SACI) test. This test showed a significant increase of the serum gastrin level from 4620 to 23,600 pg/mL at 20 s after the injection from the proper hepatic artery (PHA)

We performed a SACI test to investigate which site secretes gastrin. The superior mesenteric artery, proximal and distal sides of the splenic artery, and the proper hepatic artery were selectively catheterized and rapidly injected with calcium gluconate. Blood samples were obtained through a catheter from the right hepatic vein (RHV) and middle hepatic vein (MHV) at the following time points: pre-injection, and at 20, 40, 60, 90, and 120 s after the calcium gluconate injection. The serum gastrin concentration increased from 4620 to 23,600 pg/mL at 20 s in RHV, and from 5800 to 10,700 pg/mL at 90 s in MHV after calcium gluconate injection into the proper hepatic artery. No increase of serum gastrin levels was observed when calcium gluconate was injected into the remaining arteries (Fig. 2). Therefore, we confirmed the diagnosis of “primary” hepatic gastrinoma.

Brain and neck CT were performed to rule out multiple endocrine neoplasia type 1 (MEN-1); however, we found no abnormalities in the pituitary or parathyroid glands. The serum levels of calcium, phosphorous, prolactin, and parathyroid hormones were normal.

The tumor is in close proximity to the hilar region of the liver, especially the right branch of the portal vein, and partly involved the S4a region. Furthermore, it invades the MHV and requires combined resection of the MHV (Fig. 1b, c). His liver function was good, with a Child–Pugh Score of 5 points, score A (prothrombin rate 111%, total bilirubin 0.7 mg/dL, albumin 4.7 g/dL), indocyanine green clearance (K-ICG) of 0.247, and indocyanine green retention rate (R15) of 0%. In CT volumetry, the residual liver volume (after extended right hepatic lobectomy) was 547 mL (31%), and the predicted remnant K-ICG was 0.074, which was the lower limit of the safe range; thus, percutaneous transhepatic portal vein embolization (PTPE) of the right portal vein was required. After PTPE of the right portal vein, the residual liver volume increased to 876 mL (41.5%), and the predicted remnant K-ICG was 0.102. The excision allowance was met.

**Fig. 3:**
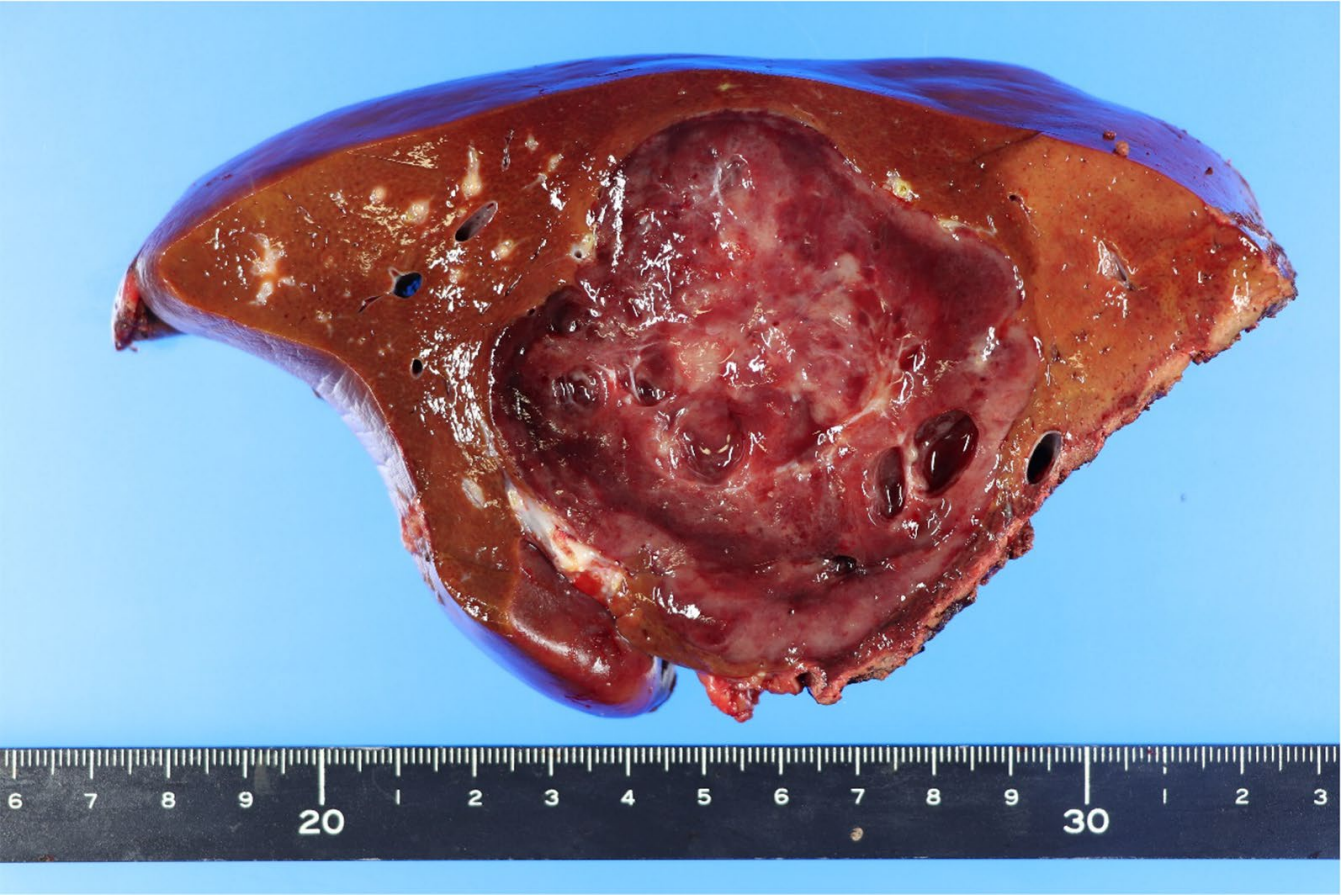
Macroscopic findings of the resected specimen. The solid mass with cystic degeneration (73 mm in diameter) was present

We performed an extended right hepatic lobectomy after 4 weeks of PTPE. In the resected liver, there was a solid mass with a cystic lesion measuring 73 mm in diameter (Fig. 3).

**Fig. 4:**
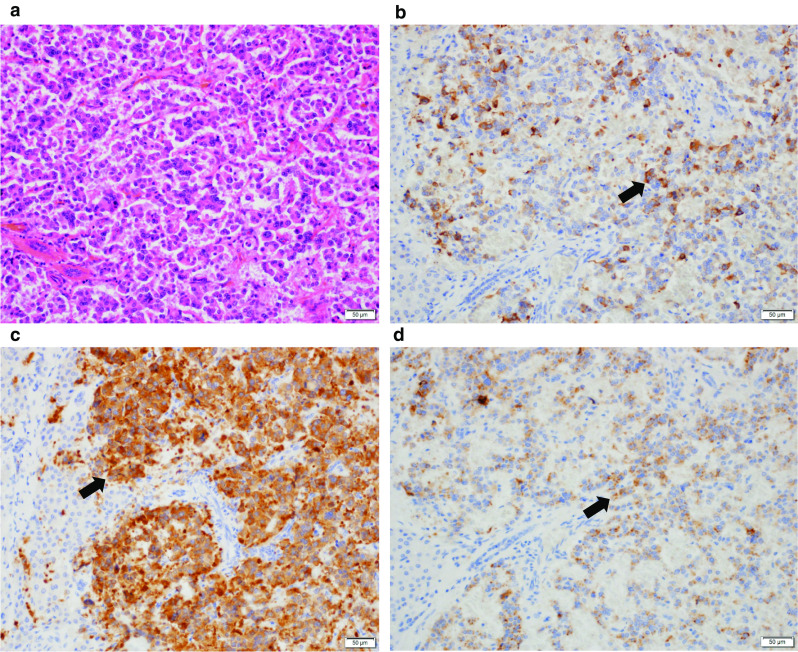
Microscopic findings of the resected specimen. **a** Hematoxylin and eosin staining of the liver mass showed a tubular, funicular, and solid increase of small cube-shaped, atypical cells. **b** Immunostaining for gastrin of the liver mass was positive (arrow). **c** Immunostaining for synaptophysin of the liver mass was positive (arrow). **d** Immunostaining for chromogranin A of the liver mass was positive (arrow)

A histopathological examination of the liver tumor revealed that it was a neuroendocrine tumor (Fig. 4a–d). The tumor cells were positive for gastrin, synaptophysin, chromogranin A, and CD56. The Ki-67 index of the tumor was 3.71%; hence, it was diagnosed as a grade 2 tumor.

The patient’s serum gastrin concentration on postoperative day 1 decreased to 65 pg/mL. He was discharged on postoperative day 12 without any comorbidity.

## Conclusions

ZES, a common presentation of most gastrinomas, was first reported in 1955, and is caused by malignant gastrin-producing neuroendocrine tumors [1]. Approximately 20–30% of patients with ZES have MEN-1. The commonly reported cure rate for gastrinomas is approximately 26% for sporadic disease [3].

Only 5.6% of all gastrinomas are located outside the triangle, which means they are extra-pancreatic, extra-duodenal, and extra-lymphatic gastrinomas [2]. A National Institutes of Health study reported that primary hepatic gastrinomas occurred in < 2% of all ZES patients [4]. Thus, primary hepatic gastrinomas are very rare.

Our patient presented with abdominal pain, vomiting, and diarrhea, which are considered characteristic symptoms of ZES. However, unfortunately, the diagnosis of ZES is becoming more difficult due to the widespread use of proton pump inhibitors (PPIs); long-term treatment with PPIs can mask the symptoms of ZES. Therefore, it may be difficult to diagnose this disease when its characteristic symptoms, such as abdominal pain, diarrhea, heartburn, and weight loss, are masked [5].

Pancreatic gastrinomas tend to be relatively large, with a mean reported diameter of 2.7–3.2 cm [3]. In contrast, 49%–80% of duodenal gastrinomas are < 1 cm in diameter [3, 5, 6]. The median size of primary hepatic gastrinomas was 4.8 cm in diameter. The sensitivity of investigative modalities, such as ultrasonography, CT, MRI, SRS, and SACI test, depends on the size of the tumor; it may be as high as 96% for neuroendocrine tumors larger than 2 cm, and it is closer to 30% for those smaller than 1 cm [3].

A gastrinoma can cause metastases even if the primary lesion is small, and the liver is a common site for metastases. It is often difficult to distinguish a primary tumor from a metastatic tumor. Most reports of gastrinomas originating in the liver have shown that the imaging and intraoperative findings of these patients revealed no lesion in any other site suspected to be the origin, and they had a high preoperative serum gastrin level that decreased immediately after resection and remained in the normal range for a certain period after the surgery [7]. Based on these findings, the liver was determined to be a primary site of these tumors.

Similar to other primary gastrinomas, the SACI test is also used to diagnose a primary hepatic gastrinoma. Gastrinomas have calcium channels; therefore, a high extracellular calcium concentration causes degranulation of the gastrinoma cells and subsequent release of gastrin. The vascular structure of a hepatic neuroendocrine tumor, such as gastrinoma, is characterized by abundant tumor vessels that lack portal blood supply and provide purely arterial nourishment [8]. Therefore, a calcium injection into vascular territories not involving gastrinomas does not cause a rise in the serum gastrin level.

To the best of our knowledge, till date, only 35 cases of primary hepatic gastrinomas have been reported, including our case (Table 1) [7–36]. Our review of the other cases reveals that the median age of patients with primary hepatic gastrinomas is 42 years, which is lower than the prevalent age (52 years) of patients with gastrinomas in the pancreatic-duodenal region [37]. No apparent sex difference was noted in the cases (male, 19 cases [54%] and female, 16 cases [46%]). Most of the reported cases were solitary tumors, and only 5 (14%) were multiple tumors. The median tumor diameter was 48 mm. In all cases, the preoperative diagnosis was ZES. SRS or SACI tests were performed for preoperative localization diagnosis in 8 cases (23%), whereas both modalities were used together in 3 cases (8%). In the SACI test, only our case had blood samples obtained from both the RHV and MHV to assess the serum gastrin levels. It was suggested that the evaluation of these factors would allow for a more reliable diagnosis of localization.

**Table 1:** Reported cases of primary hepatic gastrinoma

Author (year)	Patient	Location	Number	Size (mm)	Serum gastrin level (pg/mL)		ZES	Preoperative localization diagnosis	Performed operation	Outcome	Recurrence
					Before resection	After resection					
Gould (1981)	34 F	Right	Multiple	130	170,750	No resection	+	ND	No resection	36 M, alive	ND
Wolfe (1982)	61 F	Left	Solitary	ND	1500	82	+	−	Total gastrectomy, hepatectomy (ND)	> 120 M, alive	ND
Thompson (1983)	47 M	Right	Solitary	ND	2700	WNL	+	−	Total gastrectomy, extended right hepatectomy	13 M, alive	ND
Smith (1984)	8 M	Right	Solitary	20	893 fmol/ml (< 56)	WNL	+	−	Right anterior segmentectomy	18 M, alive	ND
Thompson (1985)	23 M	Right	Solitary	25	670	WNL	+	−	ND	24 M, alive	ND
Arnold (1989)	35 F	Both	Multiple	ND	81 pmol/L (< 40)	WNL	+	−	Liver transplantation	38 M, alive	ND
Larriva-Sahd (1992)	13 M	Right	Solitary	32	27,175	WNL	+	−	Local excision	24 M, alive	ND
Goletti (1992)	30 M	Right	Solitary	50	572	64	+	−	Right hemihepatectomy	60 M, alive	ND
Moriura (1993)	50 F	Right	Solitary	46	ND	WNL	+	−	Anterior inferior segmentectomy	24 M, alive	ND
Inoue (1993)	43 F	Left	Solitary	20	238	60–100	ND	ND	ND	60 M, alive	ND
Kanakia (1994) Krishnamurthy (1996)	9 M	Right	Solitary	60	1800	38–103	+	−	Selective vagotomy with wedge resection gastrojejunostomy	36 M, alive	ND
Wu (1997)	27 F	ND	ND	ND	289	45–76	ND	ND	ND	81 M, alive	ND
	35 M	ND	ND	ND	1607	44–56	ND	ND	ND	16 M, alive	ND
	42 M	ND	ND	ND	478	106–200	ND	ND	ND	95 M, alive	+
Tiomny (1997)	50 M	Left	Solitary	70	150	WNL	+	+(SRS)	Left hemihepatectomy	18 M, alive	ND
Kehagias and Smyrniotis (1999)	57 M	Left	Solitary	180	400	50.4	+	−	Left hemihepatectomy	14 M, alive	ND
Chien (2001)	27 F	Right	Multiple	150	1224	WNL	+	−	Right lobectomy, partial segmentectomy	42 M, alive	ND
Diaz (2003)	13 M	Left	Solitary	70	1141	30	+	+(SRS)	Left lateral hepatectomy	48 M, alive	ND
Delgado (2004)	29 F	Left	Solitary	70	1149	WNL	+	+(SRS)	Left hepatectomy	36 M, alive	ND
Ulusan (2005)	46 F	Right	Solitary	120	190	ND	+	−	ND	ND	ND
Shibata (2006)	50 M	Right	Solitary	45	1500	WNL	+	+(SACI)	Extended right hemihepatectomy	60 M, alive	ND
Ishikawa (2008)	44 F	Right	Solitary	40	1500	WNL	+	−	Enucleation (S8/4)	12 M, alive	ND
Rascarachi (2009)	51 M	Left	Solitary	100	114	19	+	−	Left hemihepatectomy	24 M, alive	ND
Kuiper (2009)	39 M	Left	Solitary	ND	889 ng/L	347 ng/L	+	−	Segmentectomy of S2	> 192 M, alive	+
Evans (2010)	46 F	Left	Solitary	40	3120	41	+	−	Left lateral segmentectomy	> 2 M, alive	ND
Tsalis (2011)	56 M	Left	Solitary	10	1688	19	+	−	Left hemihepatectomy	20 M, alive	ND
Otsuka (2012)	35 F	Both	Multiple	63	3800	67	+	−	Posterior segmentectomy	81 M, alive	+
Harvey (2012)	48 M	Right	Solitary	65	288	WNL	+	−	Extended right hemihepatectomy	72 M, alive	ND
Schroeder (2015)	51 F	Right	Solitary	34	> 5000	77	+	−	Caudate lobe resection	6 M, alive	ND
Lu (2012)	51 F	Right	Solitary	34	36,357	38	+	− (Exploratory laparotomy)	ND	6 M, alive	ND
Naoe (2012)	77 F	Right	Multiple	19	41,000	WNL	+	+(SACI)	Right lateral segmentectomy	12 M, alive	ND
Ogawa (2015)	28 M	Right	Solitary	12	846	WNL	+	+(SACI)	ND	6 M, alive	ND
Hagi (2017)	57 F	Right	Solitary	23	12,037	< 50	+	+(SACI)	Anterior segmentectomy, duodenectomy	48 M, alive	ND
Pipek (2018)	19 M	Both	Solitary	195	6709	40.5	+	−	Left trisegmentectomy	12 M, alive	ND
Our case	29 M	Right	Solitary	78	3580	65	+	+	Extended right hemihepatectomy		

*M* month, *ND* no description, *WNL* within normal limits

Surgical resection was performed in all, but one case, and the prognosis was good. Except for 3 cases (1 enucleation case, 1 partial resection case, and 1 liver transplantation case), anatomic resection was performed. There were no cases of lymph node dissection. Some clinicians argue that lymph node dissection should be performed in pancreatic, duodenal, and lymphatic gastrinomas, but the significance of lymph node dissection is unclear in primary hepatic gastrinomas. Thus, future studies on the presence and pattern of recurrence with long-term follow-up are warranted.

The median observation period was 24 months, and all patients survived in that period. Recurrence was observed in 4 cases, and the site of recurrence was the residual liver in 3 cases and lymph node in 1 case. The fasting serum gastrin level is a useful marker of recurrence; however, caution is necessary when using it to monitor recurrence because it is known to be high, even after the administration of PPIs [35].

A few important points should be noted when following up patients with gastrinomas. First, the median time of recurrence in patients with all types of gastrinomas was 5 years. The removal of a metastatic source can result in the normalization of symptoms and biochemical indices in the early postoperative period. In addition, a recurrence of a missed primary tumor can occur after a significant period following the initial operation [38]. Therefore, in this case, a long-term follow-up period is necessary to ensure that there is no primary site.

We reported a rare case of primary hepatic gastrinoma. There is a lack of novelty in terms of clinical course and treatment in our report, however there have been no reports of long-term follow-up. Therefore, we intend to continue to follow up our case. It is difficult to diagnose the “primary site” of such gastrinomas. For locating a functional endocrine tumor, tests such as SACI and SRS are useful; however, their results should be interpreted with caution. We performed a surgical excision of the tumor in our patient, which led to his recovery. We propose that some points must be considered while treating such patients. First, there must be a clear clinical and biochemical evidence of ZES. Second, appropriate pre- and intra-operative searches for an occult primary tumor, especially in the duodenum, must be carried out. Last, a long-term clinical, biochemical, and radiologic follow-up must be performed.

## Data Availability

Not applicable.

## Abbreviations

ZES: Zollinger–Ellison syndrome
CT: Computed tomography
MEN-1: Multiple endocrine neoplasia type 1
MRI: Magnetic resonance imaging
PPI: Proton pump inhibitor
ACI test: Selective arterial calcium injection test
SRS: Somatostatin receptor scintigraphy
PTPE: Percutaneous transhepatic portal vein embolization

## References

[1] Zollinger RM, Ellison EH (1955). Primary peptic ulceration of the jejunum associated with islet cell tumors of pancreas. Ann Surg.

[2] Wu PC, Alexander HR, Bartlett DL, Doppman JL, Fraker DL, Norton JA (1997). A prospective analysis of the frequency, location, and curability of ectopic (nonpancreaticduodenal, nonnodal) gastrinoma. Surgery.

[3] Fendrich V, Langer P, Waldmann J, Bartsch DK, Rothmund M (2007). Management of sporadic and multiple endocrine neoplasia type 1 gastrinomas. Br J Surg.

[4] Roy PK, Venzon DJ, Shojamanesh H, Abou-Saif A, Peghini P, Doppman JL (2000). Zollinger–Ellison syndrome. Clinical presentation in 261 patients. Medicine.

[5] Norton JA, Fraker DL, Alexander HR, Venzon DJ, Doppman JL, Serrano J (1999). Surgery to cure the Zollinger–Ellison syndrome. N Engl J Med.

[6] Norton JA (1999). Intraoperative methods to stage and localize pancreatic and duodenal tumors. Ann Oncol.

[7] Kuiper P, Biemond I, Verspaget H, Lamers C (2009). A case of recurrent gastrinoma in the liver with a review of “primary” hepatic gastrinomas. BMJ Case Rep.

[8] Ogawa S, Wada M, Fukushima M, Shimeno N, Inoue S, Chung H (2015). Case of primary hepatic gastrinoma: diagnostic usefulness of the selective arterial calcium injection test. Hepatol Res.

[9] Gould VE, Banner BF, Baerwaldt M (1981). Neuroendocrine neoplasms in unusual primary sites. Diagn Histopathol.

[10] Wolfe MM, Alexander RW, McGuigan JE (1982). Extrapancreatic, extraintestinal gastrinoma: effective treatment by surgery. N Engl J Med.

[11] Thompson JC, Lewis BG, Wiener I, Townsend CM (1983). The role of surgery in the Zollinger–Ellison syndrome. Ann Surg.

[12] Smith AL, Auldist AW (1984). Successful surgical resection of an hepatic gastrinoma in a child. J Pediatr Gastroenterol Nutr.

[13] Thompson NW, Vinik AI, Eckhauser FE, Strodel WE (1985). Extrapancreatic gastrinomas. Surgery.

[14] Arnold JC, O’Grady JG, Bird GL, Calne RY, Williams R (1989). Liver transplantation for primary and secondary hepatic apudomas. Br J Surg.

[15] Larriva-Sahd J, Angeles-Angeles A, Hernandez-Pando R, Munoz Fernandez L, Rondan A, Estevez H (1992). Ultrastructural and immunocytochemical study of a primary gastrinoma of the liver. Ultrastruct Pathol.

[16] Goletti O, Chiarugi M, Buccianti P, Tortora A, Castagna M, Del Guerra P (1992). Resection of liver gastrinoma leading to persistent eugastrinemia. Case report. Eur J Surg.

[17] Moriura S, Ikeda S, Hirai M, Naiki K, Fujioka T, Yokochi K (1993). Hepatic gastrinoma. Cancer.

[18] Inoue Y, Nakamura H, Mizumoto S, Yamasaki K (1994). Unusual manifestation of rectal duplication cyst: a case report. Radiat Med.

[19] Kanakia RR, Sawant PD, Nanivadekar SA, Vishwanath N, Rajagopalan K, Shroff CP (1994). Primary gastrinoma of the liver. Indian Pediatr.

[20] Krishnamurthy SC, Duta V, Pai SA, Kane SV, Jagannath P, Desouza LJ (1996). Primary carcinoid tumor of the liver: report of four resected cases including one with gastrin production. J Surg Oncol.

[21] Tiomny E, Bril S, Baratz M, Messer G, Greif F, Moshkowitz M (1997). Primary liver gastrinoma. J Clin Gastroenterol.

[22] Kehagias D, Moulopoulos L, Smirniotis V, Pafiti A, Ispanopoulos S, Vlahos L (1999). Imaging findings in primary carcinoid tumour of the liver with gastrin production. Br J Radiol.

[23] Smyrniotis V, Kehagias D, Kostopanagiotou G, Tripolitsioti P, Paphitis A (1999). Primary hepatic gastrinoma. Am J Gastroenterol.

[24] Chien RN, Chen TC, Chiu CT, Tsai SL, Jen LB, Liaw YF (2001). Case report: primary calcified gastrinoma of the liver. Dig Dis Sci.

[25] Diaz R, Aparicio J, Pous S, Dolz JF, Caldero V (2003). Primary hepatic gastrinoma. Dig Dis Sci.

[26] Delgado J, Delgado B, Sperber AD, Fich A (2004). Successful surgical treatment of a primary liver gastrinoma during pregnancy: a case report. Am J Obstet Gynecol.

[27] Ulusan S, Kizilkilic O, Yildirim T, Tercan F, Bolat F, Yildirim S (2005). Primary hepatic carcinoid tumor: dynamic CT findings. Abdom Imaging.

[28] Shibata C, Naito H, Funayama Y, Fukushima K, Takahashi K, Unno M (2006). Diagnosis and surgical treatment for primary liver gastrinoma: report of a case. Dig Dis Sci.

[29] Ishikawa Y, Yoshida H, Mamada Y, Taniai N, Matsumoto S, Bando K (2008). Curative resection of primary hepatic gastrinoma. Hepatogastroenterology.

[30] Rascarachi G, Siera M, Hernando M, Diez R, Arias L, Jorquera F (2009). Primary liver carcinoid tumour with a Zollinger–Ellison syndrome—an unusual diagnosis: a case report. Cases J.

[31] Evans JT, Nickles S, Hoffman BJ (2010). Primary hepatic gastrinoma: an unusual case of Zollinger–Ellison syndrome. Gastroenterol Hepatol.

[32] Tsalis K, Vrakas G, Vradelis S, Dimoulas A, Pilavaki M, Papaemmanouil S (2011). Primary hepatic gastrinoma: report of a case and review of literature. World J Gastrointest Pathophysiol.

[33] Ohtsuka M, Nakajima M, Kimura F, Shimizu H, Yoshidome H, Miyazaki M (2012). A case of gastrinoma probably originating from the liver. Nihon Rinsho Geka Gakkai Zasshi.

[34] Naoe H, Iwasaki H, Kawasaki T, Ozaki T, Tsutsumi H, Okuda A (2012). Primary hepatic gastrinoma as an unusual manifestation of Zollinger–Ellison syndrome. Case Rep Gastroenterol.

[35] Hagi T, Hosoda Y, Komoto I, Uemoto S, Hijioka S, Taki Y (2017). A primary hepatic gastrinoma accompanied by hyperplasia of multi-nodular Brunner’s glands. Surg Case Rep.

[36] Pipek LZ, Jardim YJ, de Mesquita GHA, Nii F, de Almeida Medeiros KA, Carvalho BJ (2018). Large primary hepatic gastrinoma in young patient treated with trisegmentectomy: a case report and review of the literature. World J Hepatol.

[37] Izumiyama H, Hirata K (2008). Imaging diagnosis of pancreatic endocrine tumor -1 CT MRI, Octreo Scan. Pancreas.

[38] Harvey A, Pasieka JL, Al-Bisher H, Dixon E (2012). Primary hepatic gastrinoma causing Zollinger–Ellison syndrome: a rare and challenging diagnosis. Cancers.

